# Reliability and acceptability of the multiple mini-interviews for selection of residents in cardiology: Student response

**DOI:** 10.30476/JAMP.2020.86698.1260

**Published:** 2021-10

**Authors:** SAKERIYA FARAH, KAMAL DEWAN, NAZIFA BEGUM, INAAM MUNEER MIAN, RAMEEZ SHAHZAD

**Affiliations:** 1 Barts and the London School of Medicine and Dentistry, Garrod Building, Turner Street, Whitechapel, London, E1 2AD, United Kingdom; 2 King’s College London, Guy's Campus, Great Maze Pond, London SE1 1UL

## Dear Editor

This is a letter in response to the recent study published by Burgos LM et al ( 1 ). Whilst many studies on the use of the multiple mini-interview (MMI) in medical school admissions exist, there has been a paucity of studies investigating the use of the MMI in residency programmes, with this study being the first, to the best of our knowledge. We, therefore, would like to commend the authors for their valuable data. 

Whilst we agree with the notion that the MMI is a more reliable and feasible tool in assessing the candidates, there are a few limitations of this format that we would like to add to the comprehensive analysis presented in this paper. 

The relationship between a candidate’s personality and his/her MMI score is something that ought to be considered. Studies have shown that the personality of each individual can significantly affect his/her performance in MMI stations ( 2 - 5 ). A study conducted by Jerant et al. ( 2 ) on 444 medical applicants showed that extroversion was linked with a high MMI score and, thus, a better performance compared to candidates who scored low on the extroversion tests. This phenomenon has been reported in later studies, by Griffin and Wilson et al. ( 3 ), and Oliver et al. ( 4 ), respectively. In addition, anxiety and stress have been shown to be more common in introverts compared to extroverts ( 6 ). Medical students with introverted personality types may, thus, experience stress and anxiety more than their extroverted counterparts. This emotional feeling is heightened during the MMI process. One-third of applicants to the University of Dundee Medical School indicated they felt the MMI was more stressful than traditional panel interviews ( 7 ). Similarly, 44% of the applicants to the School of Medicine, UCLA, indicated that they found the MMI stressful ( 8 ). We can, thus, infer based on this evidence that a personality type other than extroversion, coupled with the common feeling of anxiety and stress, may significantly affect the performance of applicants undertaking the MMI format. Further investigation ocarried out on the effect of these two factors on MMI scores will allow us to comment on the reliability of this method of assessment more accurately. 

Lastly, although MMIs do not appear biased against applicants on the basis of age, gender, or socio-economic status, studies have highlighted that certain ethnic and social backgrounds are performed less well ( 5 ). A study conducted by ME Kelly et al. ( 9 ) indicated a strong correlation between MMI scores and the English language proficiency (IELTS). Applicants from Non-EU backgrounds and those who did not have English as their first language scored significantly lower than their EU and English-speaking counterparts. Whilst this factor may not be of high significance in countries where ethnic diversity rates are low, it will certainly affect the scores of applicants in ethnically diverse cities across the globe, such as London ( 10 ). We recommend that the authors also should recognize the significance of this factor, and a replication of this study is required in various parts of the globe in order to observe the social effect of the culture and region on the reliability of this assessment modality.

We agree that the MMI is feasible, valid and more reliable than the other types of formats already used to interview the candidates, and the overwhelming evidence presented in the systematic review by L Rees et al. ( 5 ) supports this view. However, considering the limitations mentioned, it is important to note that the reliability of this method is contingent on a carefully designed format.

## References

[1] Burgos LM, De Lima  A, Parodi J, Costabe JP, Ganiele MN, Durante E, et al ( 2020). Reliability and acceptability of the multiple mini-interview for selection of residents in cardiology. Journal of Advances in Medical Education & Professionalism.

[2] Jerant A, Griffin E, Rainwater J, Henderson M, Sousa F, Bertakis K, et al ( 2012). Does Applicant Personality Influence Multiple Mini-Interview Performance and Medical School Acceptance Offers?. Acad Med.

[3] Griffin B, Wilson I ( 2012). Associations between the big five personality factors and multiple mini-interviews. Advances in Health Sciences Education.

[4] Oliver T, Hecker K, Hausdorf P, Conlon P ( 2014). Validating MMI scores: are we measuring multiple attributes?. Advances in Health Sciences Education.

[5] Rees E, Hawarden A, Dent G, Hays R, Bates J, Hassell A ( 2016). Evidence regarding the utility of multiple mini-interview (MMI) for selection to undergraduate health programs: A BEME systematic review: BEME Guide No. 37. Med Teach.

[6] Rentala S, Aladakatti R ( 2019). Academic Stress among Indian Adolescent Girls. Journal of Education and Health promotion.

[7] Dowell J, Lynch B, Till H, Kumwenda B, Husbands A ( 2012). The multiple mini-interview in the UK context: 3 years of experience at Dundee. Med Teach.

[8] Uijtdehaage S, Doyle L, Parker N ( 2011). Enhancing the Reliability of the Multiple Mini-Interview for Selecting Prospective Health Care Leaders. Acad Med.

[9] Kelly M, Dowell J, Husbands A, Newell J, O‘Flynn S, Kropmans T, et al ( 2014). The fairness, predictive validity and acceptability of multiple mini interview in an internationally diverse student population- a mixed methods study. BMC Medical Education.

[10] White ethnic Britons in minority in London [Internet] Financial Times. 2011 [cited 19 May 2020];. http://www.ft.com/cms/s/0/4bd95562-4379-11e2-a48c-00144feabdc0.html#axzz2EmHkrZcr.

